# Supplementary material for: The genomes of precision edited cloned calves show no evidence for off-target events or increased de novo mutagenesis
Source: BMC Genomics. 2021 Jun 17;22:457. doi: 10.1186/s12864-021-07804-x (PMC8212539; doi:10.1186/s12864-021-07804-x)
Supplement: Supplementary file 2 — Additional file 2: Table S2 Structural variants (SVs) identified in the gene-edited cell line (CC14) and gene-edited cloned calves (1805 and B071) using DELLY with the parental cell line (BEF2) and non-edited cloned calves (1802, 1803 and 1804) as reference samples. Table S3 Number of variants remaining after each filter to determine their presence in control calves (1802/1803/1804), but absence in parental cell line BEF2, and presence in gene edited samples (CC14/1805/B071), but absence in BEF2. Variants were kept if they (1) were called as heterozygous or homozygous alternate by GATK HaplotypeCaller in group, but homozygous reference in BEF2, (2) had a map quality of 60, and (3) called as homozygous reference in BEF2 and had an alternate allele depth of 0. Table S4 Number of variants remaining after each filter to determine their presence in parental cell line BEF2, but absence in control calves (1802/1803/1804), and presence in BEF2, but absence in gene edited samples (CC14/1805/B071). Variants were kept if they (1) were called as heterozygous or homozygous alternate by GATK HaplotypeCaller in BEF2, but homozygous reference in group samples, (2) had a map quality of 60, and (3) called as homozygous reference in group samples and had an alternate allele depth of 0. Table S5 Description of PCR primer pairs designed to investigate the on-target site and plasmid integration. Figure S1 First two principal components (PC) for non-edited control calves (1802/1803/1804), gene edited samples (CC14/1805/B071), and the parental cell line BEF2 plotted against each other revealed no clustering by treatment group. Figure S2 Uncropped, full-length versions of the gels presented in ‘Fig. 3’. [file 12864_2021_7804_MOESM2_ESM.docx]

# The genomes of precision edited cloned calves show no evidence for off-target events or increased *de novo* mutagenesis

# Supplementary Materials

Swati Jivanji^1*^, Chad Harland^2^, Sally Cole^3^, Brigid Brophy^3^, Dorian Garrick^1^, Russell Snell^4^, Mathew Littlejohn^1,2^, Götz Laible^3,5,6^

^1^ School of Agriculture and Environment, Massey University, Palmerston North, New Zealand

^2^Livestock Improvement Corporation, Newstead, New Zealand

^3^AgResearch, Ruakura Research Centre, Hamilton, New Zealand

^4^School of Biological Sciences, University of Auckland, Auckland, New Zealand

^5^School of Medical Sciences, University of Auckland, Auckland, New Zealand

^6^Maurice Wilkins Centre for Molecular Biodiscovery, Auckland, New Zealand

*Corresponding author

Email: swati.jivanji.1@uni.massey.ac.nz

Table S2 Structural variants (SVs) identified in the gene-edited cell line (CC14) and gene-edited cloned calves (1805 and B071) using DELLY with the parental cell line (BEF2) and non-edited cloned calves (1802, 1803 and 1804) as reference samples

|  | **CC14** | **1805** | **B071** |
| --- | --- | --- | --- |
| BEF2 as reference sample | 27 | 38 | 32 |
| 1802, 1803 and 1804 added as reference samples | 1 | 5 | 5 |
| SVs common between gene-edited samples | 0 | 0 | 0 |

Table S3 Number of variants remaining after each filter to determine their presence in control calves (1802/1803/1804), but absence in parental cell line BEF2, and presence in gene edited samples (CC14/1805/B071), but absence in BEF2. Variants were kept if they (1) were called as heterozygous or homozygous alternate by GATK HaplotypeCaller in group, but homozygous reference in BEF2, (2) had a map quality of 60, and (3) called as homozygous reference in BEF2 and had an alternate allele depth of 0.

| Group | Heterozygous or homozygous alternate in group samples but homozygous reference in BEF2 | Quality-filtered variants | | Alternate allele depth = 0 in BEF2 |
| --- | --- | --- | --- | --- |
| 1802/1803/1804 | 972 | 256 | 60 | |
| CC14/1805/B071 | 1338 | 452 | 253 | |

Table S4 Number of variants remaining after each filter to determine their presence in parental cell line BEF2, but absence in control calves (1802/1803/1804), and presence in BEF2, but absence in gene edited samples (CC14/1805/B071). Variants were kept if they (1) were called as heterozygous or homozygous alternate by GATK HaplotypeCaller in BEF2, but homozygous reference in group samples, (2) had a map quality of 60, and (3) called as homozygous reference in group samples and had an alternate allele depth of 0.

| Group | Homozygous reference in group samples but heterozygous or homozygous alternate in BEF2 | Quality-filtered variants | | Alternate allele depth = 0 in group samples |
| --- | --- | --- | --- | --- |
| 1802/1803/1804 | 2256 | 759 | 80 | |
| CC14/1805/B071 | 2073 | 622 | 82 | |

Table S5 Description of PCR primer pairs designed to investigate the on-target site and plasmid integration

|  | Sequence | Melting Temp ( ֯C ) | PCR product size | Position |
| --- | --- | --- | --- | --- |
| **Long-range PCR primers** | | | | |
| Forward primer | GTGCCACTGACATGTAGCAAAG | 60.8 | 8,860bp | BTA5:57,340,856-57,349,715bp |
| Reverse primer | CCCTCCTCAGTCCTTACCAGTA | 59.6 |  |  |
| **Vector integration PCR primers** | | | | |
| **Set 1** |  |  |  |  |
| Forward primer | TGACGTTGGAGTCCACGTTC | 62.1 | 757bp | gRNA/Cas9 plasmid:6,263-7,019bp |
| Reverse primer | TCTTCGGGGCGAAAACTCTC | 63.9 |  |  |
| **Set 2** |  |  |  |  |
| Forward primer | AGATCAGTTGGGTGCACGAG | 61.3 | 690bp | gRNA/Cas9 plasmid:6,939-7,628bp |
| Reverse primer | TGACTCCCCGTCGTGTAGAT | 60.5 |  |  |
| **Internal control PCR primers** | | | | |
| Forward primer | ATGTTAGGTGCAGGTGGAGC | 60.1 | 519bp | BTA2:110,817,757-110,818,275bp |
| Reverse primer | GCTTCCCACCTTGACCTCTC | 61.2 |  |  |

Supplementary Figure 1: First two principal components (PC) for non-edited control calves (1802/1803/1804), gene edited samples (CC14/1805/B071), and the parental cell line BEF2 plotted against each other revealed no clustering by treatment group.


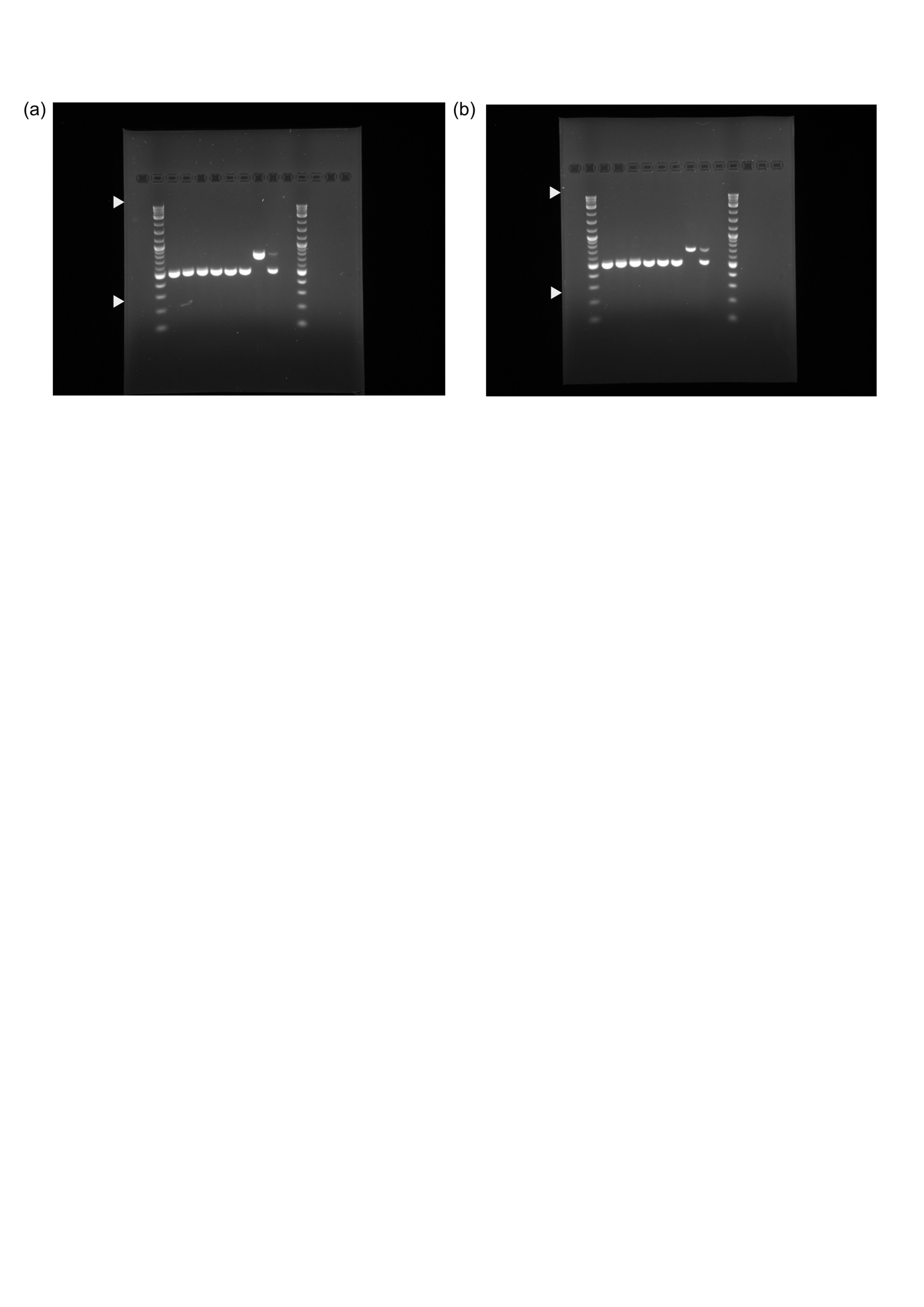


*Supplementary Figure 2: Uncropped, full-length versions of the gels presented in ‘Fig 3’. (a) and (b) correspond to Figures 3(a) and 3(b), where the white arrows indicate where the gel image was horizontally cropped. Images were also cropped vertically to remove the background.*
